# Gastro-Enterotomy, with Report of a Case1Read before the Fifteenth Annual Meeting of the Iowa State Veterinary Medical Association, Cedar Rapids, Iowa, January 14 and 15, 1903.

**Published:** 1903-04

**Authors:** J. Miller

**Affiliations:** Ottumwa, Ia.


### GASTRO-ENTEROTOMY, WITH REPORT OF A CASE.[1]

By J. Miller, V.S.,
OTTUMWA, IA.

The triumphs in this field of human surgery and the success attending this operation in some of the domestic animals would warrant its more general application. Gastro-enterotomy implies an incision

[1] Read before the Fifteenth Annual Meeting of the Iowa State Veterinary Medical Association, Cedar Rapids, Iowa, January 14 and 15, 1903.

of the intestine through an opening in the abdominal wall. It is the least complicated of all intestinal surgery, but the marked intolerance of some of our patients to any operation of this character demands great skill on the part of the operator in the technical part of the work. We should spare no pains in making ourselves acquainted with the most improved methods, and should by practice make ourselves familiar with the technique. This familiarity can easily be acquired, as we have frequent opportunities to hold post-mortems. It is said a fairly good method skilfully performed is better than the best method clumsily performed. A thorough knowledge of the anatomy is essential to secure the best results. Any ignorance as to the muscles and their disposition, the course of important vessels and nerves, and the arrangement of the intestines and their relation to the other organs of the abdomen, will not only make the operation more difficult, but also more hazardous.

Gastro-enterotomy is indicated in the following conditions: Coprostasis, foreign bodies in the intestines, and intestinal dilatation. Undoubtedly many lives have been sacrificed from each of these conditions that might have been saved by proper surgical interference. The diagnosis of diseased conditions within the abdominal cavity is not always easy, and every precaution should be taken to prevent arriving at erroneous conclusions. The history of the case should be thoroughly inquired into, a careful examination should be made, and not until proper medication has failed should we resort to any operation. When, however, all other agencies have failed and we are quite sure of the correctness of our diagnosis, and have good reason to believe that the life of the patient can be saved by the use of proper surgical measures, it is a wrong and a shame to fail of our duty and abandon our patient to fate.

Preparation of the Patient. Frequently there is not much time for this, as the suffering is so great and the demand for relief so urgent. The importance of dieting for a day or two previous to operating should not be overlooked, as an empty condition of the stomach and bowels greatly enhances a speedy recovery. It not infrequently happens that the illness of the patient and consequent inappetence has secured this condition. Hospitals are seldom at our command, and in many instances it is difficult to secure a suitable place to operate. A clean place should be selected as free from dust and dirt as possible, and just before operating should be sprinkled with an antiseptic solution.

Having determined on the site of the opening, it should be clipped, shaved, and disinfected. But few instruments are needed in enterotomy, but these should be in readiness before casting the animal and administering the anæsthetic. A couple of scalpels, artery forceps, scissors, a few assorted needles with suitable thread are all that are required. These, with a supply of gauze or muslin and a can of iodoform should be placed within easy reach of the operator. It is important in operations of this character to observe the following considerations with care:

1. The avoidance of all unnecessary hemorrhage.
2. The prevention of the escape of any irritating matter into the abdominal cavity.
3. The union of the divided borders so that they will remain properly joined and result in perfect repair.
4. The avoidance of any unnecessary shock or irritating influence.

The first indication is met by the avoidance of any incision through the lines of an established course of vessels and by the use of needles which do not possess cutting edges. To meet the second indication requires a great deal of care. If the nature of the case will allow, the contents of the viscus should be pushed aside before the incision is commenced. If the length of the mesentery will permit, it is advisable to bring the bowel out on the surface of the abdomen. Should this be impossible, the serous surface can be protected by packing around gauze or muslin saturated with a mild, warm antiseptic fluid. Despite every precaution, occasionally there will escape some of the contents of the intestine into the abdominal cavity. A small quantity can be sponged or wiped up, but if a considerable quantity has escaped and becomes diffused, it is best to irrigate with 6 to 10 per cent. saline solution at a temperature of 112° F. In opening up the abdomen, if the incision is made through the linea alba, the hemorrhage is so slight that no ligaturing is necessary. In making the incision at other points there is more danger of hemorrhage, and not infrequently one or more vessels will have to be tied. This should be done before the peritoneum is incised.

The incision in the small intestines should be made at a point opposite the attachment of the mesentery and parallel with the long axis of the bowel. Any tearing or separation of the mesentery from the bowel should be avoided, as it lowers the vitality and diminishes the probability of repair. In operating on the colon the incision may be either transverse or longitudinal as best suits the convenience of the operator. When chronic dilatation exists it is advisable to remove an elliptical piece, suturing the intestine to the proper size. In fulfilling the third condition sutures of various forms and methods of application are employed, the aim of all being to bring the serous, muscular, and submucous coats in contact and maintain them until perfect union is established.

The fourth indication is very important, especially if the operation be prolonged and tedious. Unwarranted rough handling, dull scalpels, and other imperfect instruments, too large or too small an incision, and all other imperfect conditions tend to increase the shock.

Intestinal Suture. We have many varieties of intestinal sutures to choose from, but some are too complicated to be practical. My aim, therefore, will be to describe only those having the sanction of practicability. Needles that displace and do not cut the tissues in the passing are the ones employed in intestinal sewing, the common cambric needle being a good illustration of the kind. Catgut or silk sutures are used, but the latter is preferable to the former, as it is not so liable to stretch and allow the parts to separate before a perfect union has taken place. A size suitable to the work is selected and sterilized, the iron being preferable when the suturing has to be done in the abdomen, where it is difficult to make sure of the exact location of each stitch, on account of a poor light or other conditions which obscure the operation. The strength of the suture should be tested lest it should break when applied, confusing the surgeon and delaying the operation. Much stronger thread is required in suturing the stomach of the ox than the intestines of this or other animals, on account of the size and weight of this organ and its vigorous movements.

The sutures should include the serous, muscular, and submucous coats. The borders of the incision should be turned in and the serous surfaces brought together and transfixed by either one of the following methods:

The Continuous Suture. This is a very useful and a very practical suture in joining the borders of the incision, as it is very easily and quickly applied, the stitches being placed about two lines apart, in this respect differing from cutaneous sewing.

The Gely Suture. A long thread is secured, armed with a needle at each end. The needles are inserted about half an inch from the angle of the wound and carried along the tissues of the bowel for about a sixth of an inch, and brought out on the same level so as to appear on the peritoneal surface. The needles are crossed and passed as before. If a knot is made at each crossing, slipping is prevented. This suture, although a good one, is not so well understood and cannot be as promptly applied.

The Cushing Suture. In this suture, as in all others, the serous, muscular, and submucous coats are included. The thread is knotted and the needle inserted in a longitudinal direction for about two lines, and is brought out on the serous surface. It is now carried to the opposite side, and, at the same distance from the border of the incision, is passed as before, being carried back again and the process repeated. When the thread is drawn tight the wound is closed and the stitches are concealed.

LEMBERT'S SUTURE. This method is one that is commonly used and is quite reliable. It consists of a number of interrupted sutures which never come in contact with the contents of the alimentary canal. There is no slipping in this method, as each stitch is tied by itself. Each stitch being independent, the premature giving way of any one would not necessarily interfere with any of the rest or result in the opening of the wound, as might occur with a continuous suture.

THE CZERNY-LEMBERT SUTURE. Two rows of sutures are employed in this method, neither, however, passes through the mucous membrane. The first, being the deeper series, are placed quite close to the borders of the incision, bringing them into contact; the second, the Lembert, are placed farther from the borders of the wound and outside of the first series, thus making two rows.

THE HALSTED SUTURE. The Halsted or quilt sutures are similar to those used in the knotting of a quilt. Tying should be omitted when practicable until the sutures are placed.

The methods of closing the opening in the abdominal wall are practised: (1) That in which certain tissues are joined independently with each other, called tier suturing; (2) that in which the borders are joined as a whole, sometimes called suturing en masse. In the former the peritoneum is joined independently with a continuous suture, later the muscles, and, finally, the skin by interrupted or Lembert sutures. The wound should be thoroughly cleaned and dusted with iodoform, and before the opening is completely closed the air should be forced out by pressing the walls of the abdomen. In the latter method the borders are transfixed by using two needles passing from within outward, thus reducing to a minimum the probability of infection. Although this plan is the best, the transfixing may be done in the usual way, using but one needle, and passing from right to left, or the reverse, penetrating one side from without and the other from within. In the small animals this latter method is quite safe and just as effectual.

AFTER-TREATMENT, The patient should be kept as quiet and comfortable as possible. No solid food should be allowed for six or seven days. Small quantities of water, milk, or oatmeal solution can be given. Sometimes the pulse becomes very feeble and the strength of the patient rapidly runs down, showing that the system has been greatly disturbed by the operation. This is what is commonly called shock, and should be met by the use of stimulants hypodermically, followed later by subcutaneous injections of saline solution in extreme cases. Peritonitis is one of the most likely complications, and should be overcome by fomentation and morphine. Nutritious enemas may be used to keep up the strength. The wound may be coated with collodion, or, where practicable it may be protected by sterilized gauze held in position by a broad body-bandage. Because of unfavorable circumstances it is difficult to get union by first intention, therefore, it is wise to examine the wound on the second day, and, if need be, dress it once a day. Where there has been considerable infection of the abdominal cavity, the symptoms become alarming, it is best in such cases to open the wound and irrigate the cavity with a weak bichloride solution, 1 : 10,000. Occasionally the stitches will give way before union of the borders has taken place, demanding re-suturing.

In connection with this paper I have two cases to report:

The first was a case of coprostasis in a member of the bovine family. Strictly speaking, this case does not come within the title of this paper, but, as it bears such a close analogy, I have thought best to include it. The operation was one of rumenotomy on a dairy cow in the prime of life. The first operator has failed in securing union of either the stomach or the abdominal walls, and I was called to complete his work. Both openings were gaping at the time, and the movements of the stomach could be easily seen. The cow did not seem to be much inconvenienced and ate reasonably well of the soft food given her. The first sutures giving way before perfect union had taken place, I found it necessary to again apply them to permanently close the openings, this making the third time the parts had been sutured.

The second case was one of foreign bodies in the intestines of a pug dog. This little sufferer has been sick for about ten days. The symptoms were those of inactivity of the bowels, occasional ineffectual attempts at defecation, with languor and vomiting of all solid food and medicine for the last few days. The obstructions were found to be in the floating colon, and consisted of two intestinal concretions, spherical in shape, almost one inch and a half in diameter and made up of pieces of bones and sand cemented together with alimentary matter and as hard as a rock. He rallied from the operation in good shape, and, although quietude was enjoyed, he insisted upon going about the house, and even up stairs, and refused to be carried. Primary union in the wound was realized, and there was no suppuration except at the points where the stitches were inserted. The stitches were removed on the seventh day. By this time he was practically well, save a little complication which occurred at the time of administering the anæsthetic, viz., keratitis. No bandages were used in either case. The wound was dusted with equal parts of talcum and tannoform two or three times a day.

What I have said is based upon a limited but successful experience, and I am of the opinion that this and other operations for the relief of neoplasms, volvulus, intussusception, and strangulation should be, and I doubt not will be, performed more frequently.

